# Bevacizumab combined with platinum–taxane chemotherapy as first-line treatment for advanced ovarian cancer: a prospective observational study of safety and efficacy in Japanese patients (JGOG3022 trial)

**DOI:** 10.1007/s10147-018-1319-y

**Published:** 2018-07-20

**Authors:** Shinichi Komiyama, Kazuyoshi Kato, Yuki Inokuchi, Hirokuni Takano, Takashi Matsumoto, Atsushi Hongo, Mikiko Asai-Sato, Atsushi Arakawa, Shoji Kamiura, Tsutomu Tabata, Nobuhiro Takeshima, Toru Sugiyama

**Affiliations:** 1 Department of Gynecology, Toho University Ohashi Medical Center, 2-17-6, Meguro-ku, Tokyo, 153-8515 Japan; 2 Department of Gynecology, Cancer Institute Hospital, Tokyo, Japan; 3 Department of Biostatistics, Kitasato Academic Research Organization, Kitasato University, Tokyo, Japan; 4 Department of Obstetrics and Gynecology, The Jikei University Kashiwa Hospital, Kashiwa, Japan; 5 Department of Obstetrics and Gynecology, Ehime University School of Medicine, Matsuyama, Japan; 6 Department of Obstetrics and Gynecology 2, Kawasaki Medical School, Okayama, Japan; 7 Department of Obstetrics and Gynecology, Yokohama City University School of Medicine, Yokohama, Japan; 8 Department of Obstetrics and Gynecology, Nagoya City University Graduate School of Medical Sciences, Nagoya, Japan; 9 Department of Gynecologic Oncology, Osaka International Cancer Institute, Osaka, Japan; 10 Department of Obstetrics and Gynecology, Mie University Faculty of Medicine, Tsu, Japan; 11 Department of Obstetrics and Gynecology, Iwate Medical University, Morioka, Japan

**Keywords:** Advanced epithelial ovarian cancer, First-line chemotherapy, Bevacizumab, Paclitaxel plus carboplatin, Platinum-free interval

## Abstract

**Background:**

This was the first large-scale prospective observational Japanese study evaluating the safety and efficacy of bevacizumab combined with paclitaxel and carboplatin for newly diagnosed advanced ovarian cancer.

**Methods:**

Patients were prospectively enrolled in the primary analysis cohort if they had Stage III or IV epithelial ovarian/fallopian tube/primary peritoneal cancer and were scheduled to receive paclitaxel plus carboplatin every 3 weeks in Cycles 1–6 and bevacizumab every 3 weeks in Cycles 2–22. Primary endpoints were bevacizumab-specific adverse events and adverse events ≥ Grade 3. Secondary endpoints were progression-free survival (PFS) and the response rate.

**Results:**

Among 346 patients enrolled, 293 patients formed the primary analysis cohort. Regarding bevacizumab-specific adverse events ≥ grade 3, incidence rates of thromboembolic events (1.4%), gastrointestinal perforation (0.3%), fistula (0.7%), wound dehiscence (0%), and bleeding (0%) were very low. While incidence rates of hypertension (23.2%) and proteinuria (12.6%) were high, all such events were tolerable. No patient with prior bowel resection developed perforation or fistula. Median PFS was 16.3 months (95% CI 14.5–18.9). The response rate was 77.5% (95% CI 67.4–85.7). The response rate was 63.6% in patients with clear cell carcinoma, which tended to be better than previously reported. The median platinum-free interval was 11.5 months, and the platinum-resistant recurrence rate was 24.5%.

**Conclusions:**

Combining bevacizumab with chemotherapy was tolerable and efficacy was acceptable in Japanese patients with advanced epithelial ovarian cancer. Bevacizumab seems to reduce platinum-resistant recurrence and is promising for clear cell carcinoma.

## Introduction

In Japan, it is estimated that 9804 new patients develop ovarian cancer every year and the estimated annual number of deaths from this cancer is 4758, with its outcome being the worst among female genital tract cancers [1]. The underlying reasons are that about 90% of ovarian cancer is epithelial carcinoma, with approximately 50% of epithelial ovarian cancers being Stage III or Stage IV advanced disease at diagnosis [2].

Standard treatment for epithelial ovarian cancer (including fallopian tube cancer and primary peritoneal cancer) is doublet taxane plus platinum therapy. Various new drugs have been developed to improve the outcome of patients with advanced epithelial ovarian cancer. The molecular-targeting agent bevacizumab is a recombinant humanized monoclonal antibody directed against human vascular endothelial growth factor (VEGF). Bevacizumab inhibits VEGF signaling by blocking the binding of VEGF to its receptors and reduces tumor growth by suppressing angiogenesis in tumor tissues. Two randomized controlled studies (GOG-0218 and ICON7) have shown that combining bevacizumab with paclitaxel and carboplatin as first-line therapy followed by maintenance bevacizumab monotherapy significantly prolongs the progression-free survival time in patients with advanced epithelial ovarian cancer [3, 4]. In Japan, based on the results of the GOG-0218 study, bevacizumab was approved for advanced ovarian cancer in November 2013.

However, bevacizumab has various specific adverse effects, including gastrointestinal perforation and thromboembolism, and management of such adverse events is important when treating ovarian cancer [3, 4]. It was recently reported that there are racial differences in the safety and efficacy of cytotoxic anticancer drugs and molecular-targeting agents, with the differences seeming larger for molecular-targeting agents [5–8]. While there is concern about such racial differences for bevacizumab, there have not been any reports about use of bevacizumab in Asian patients with ovarian cancer, including Japanese patients. Therefore, we performed the present study to evaluate the safety and efficacy of bevacizumab combined with standard treatment using paclitaxel and carboplatin in Japanese patients who had advanced epithelial ovarian cancer.

## Materials and methods

### Patients

The subjects were Japanese patients aged 20 years old or older who met the following inclusion criteria: (1) patients with a diagnosis of International Federation of Gynecology and Obstetrics (FIGO) Stage III/IV epithelial ovarian cancer/fallopian tube cancer/primary peritoneal cancer based on the findings at initial debulking surgery who were scheduled to receive first-line chemotherapy, or patients with Stage III/IV epithelial ovarian cancer/fallopian tube cancer/primary peritoneal cancer by cytological diagnosis or histological and imaging diagnosis who had undergone interval debulking surgery after neoadjuvant chemotherapy and were scheduled to receive postoperative chemotherapy, (2) patients who gave informed consent to platinum-based combination chemotherapy and concomitant bevacizumab, (3) patients in whom the period from primary debulking surgery (PDS) or interval debulking surgery (IDS) to the initiation of bevacizumab was at least 28 days, (4) patients with an Eastern Cooperative Oncology Group (ECOG) performance status of 0–2, (5) patients with adequate function of the bone marrow and major organs, and (6) patients who gave written informed consent to participation in this study.

Major exclusion criteria were as follows: (1) patients with a history of bevacizumab treatment, (2) patients with gastrointestinal perforation or fistula, (3) patients who had received abdominal radiotherapy, (4) patients with severe infectious complications, (5) patients with uncontrolled hypertension, (6) patients with arterial thromboembolism (e.g., cerebral infarction or myocardial infarction), (7) patients with venous thromboembolism (e.g., deep vein thrombosis and/or pulmonary embolism), and (8) patients with proteinuria of 2+ or more.

All patients received written information about this study and submitted written consent before enrollment.

### Study design

This was a single-arm, prospective, observational study, which was conducted in the setting of routine clinical practice without any special interventions. Since the safety and efficacy of bevacizumab combined with chemotherapy have not been confirmed in Japanese patients with advanced ovarian cancer, dosage and administration, premedication, dose reduction/interruption/discontinuation, and examinations (including imaging studies) were all performed according to the GOG-0218 study in principle [3].

**Fig. 1:**
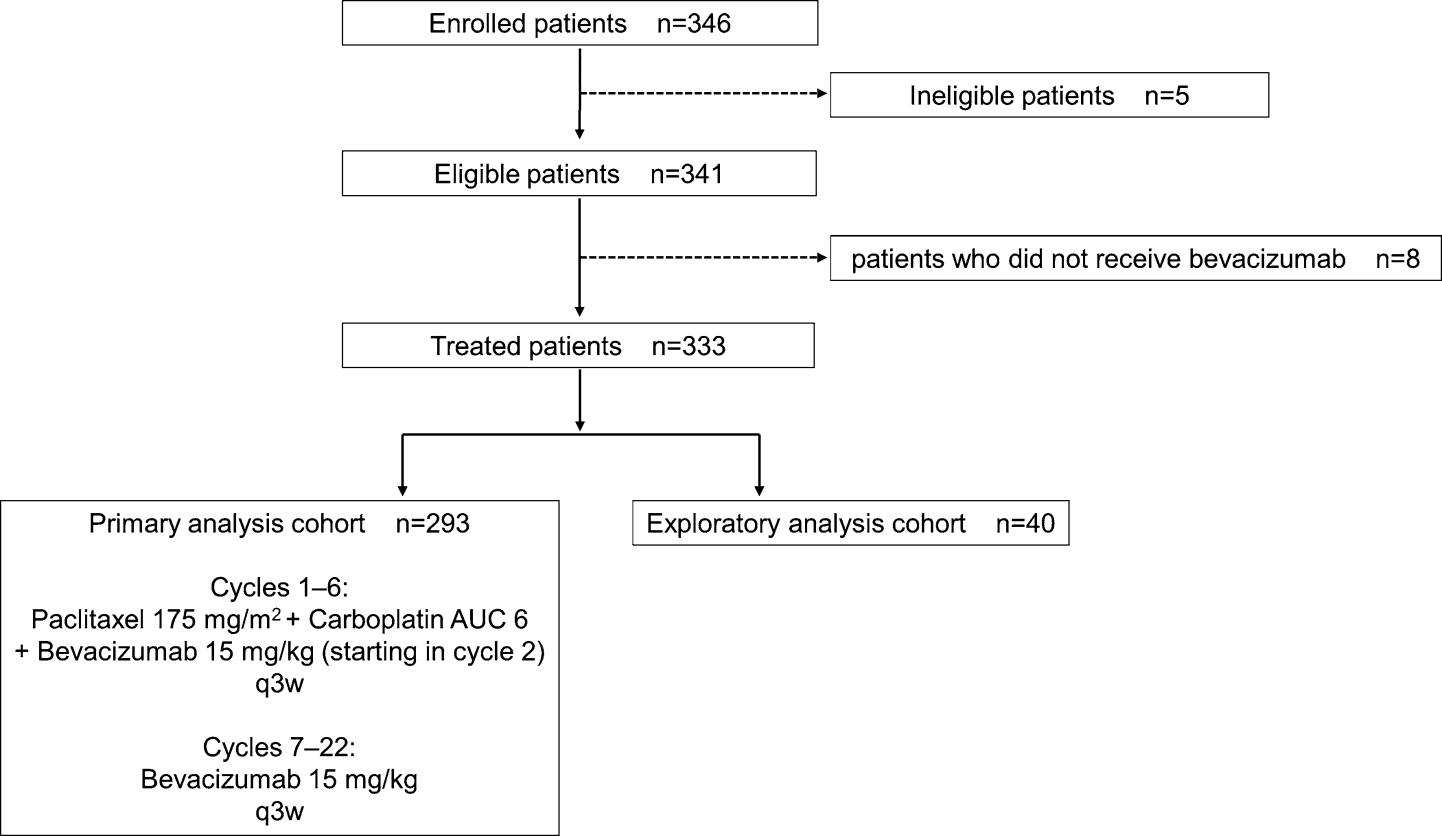
Flow chart for selection of the cohorts. The ineligible patients included three patients with venous thromboembolism, one patient with Stage IIB disease, and one patient with metastatic ovarian cancer (primary colon cancer). *AUC* area under the curve (mg/mL/min), *q3w* every 3 weeks

Patients who were scheduled to receive bevacizumab concomitantly with platinum-based combination chemotherapy after PDS or IDS were included in the observation cohort of this study before enrollment. After patients were enrolled, ineligible patients were excluded and all remaining patients were regarded as eligible patients. Of all eligible patients, patients who received at least one dose of bevacizumab were classified as treated patients. Of all treated patients, those who were scheduled to receive “bevacizumab throughout treatment” (as in the GOG-0218 study) were included in the primary analysis cohort. Specifically, they received paclitaxel (175 mg/m^2^) plus carboplatin [area under the curve (AUC) = 6 mg/mL/min] (paclitaxel plus carboplatin) every 3 weeks for cycles 1–6, with bevacizumab (15 mg/kg) being administered every 3 weeks for cycles 2–22. Patients who were enrolled but received bevacizumab concomitantly with chemotherapy other than paclitaxel plus carboplatin were also included in the exploratory analysis cohort (Fig. 1).

The primary endpoints were related to the safety of bevacizumab, including the incidence rates of bevacizumab-specific adverse events (gastrointestinal perforation, fistula, delayed wound healing, bleeding, thromboembolism, hypertension, proteinuria, congestive heart failure, and reversible posterior encephalopathy syndrome) and the incidence rate of all adverse events ≥ Grade 3 in the primary analysis cohort. The secondary endpoints were related to efficacy, including the progression-free survival time (PFS) and the response rate in the primary analysis cohort. Exploratory endpoints were the incidence rates of important bevacizumab-specific adverse events and all adverse events ≥ Grade 3 in the exploratory analysis cohort.

Adverse events were evaluated according to the Common Terminology Criteria for Adverse Events (CTCAE) Version 4.03 [9] and the frequency of the most severe grade of each event in each patient during all treatment cycles was calculated.

The tumor response and disease progression were evaluated by computed tomography (CT) or magnetic resonance imaging (MRI) according to the new Response Evaluation Criteria in Solid Tumours (revised RECIST guideline, version 1.1) [10] by each investigator. Disease progression was not judged only from changes in the serum CA-125 level. PFS was calculated as the interval from the date of enrollment to the date of detecting progressive disease (PD) or the date of all-cause death (whichever was earlier). The response rate was calculated as the percentage of patients in the analysis cohort with a measurable lesion in whom the best overall response according to RECIST was a complete response (CR) or partial response (PR). The platinum-free interval (PFI) was defined as the period from the last date of administration of a platinum-containing drug to the date when disease progression was observed by imaging.

### Statistical analysis

PFS and PFI were estimated using the Kaplan–Meier method. The standard error was calculated by the Greenwood formula, and the confidence interval of the median survival time was calculated with the method of Brookmeyer and Crowley. The confidence interval (CI) of the response rate was calculated using the Clopper–Pearson method.

If 300 patients were available for analysis, it was calculated that there was a probability ≥ 95% of detecting at least one patient who had an adverse event with the true incidence rate of ≥ 1.0%. Therefore, 300 patients were enough to evaluate safety adequately. It was also assumed that PFS evaluated by the investigators in the present study would be similar to PFS in the bevacizumab throughout treatment group of the GOG-0218 study. Accordingly, if 300 patients were enrolled (with a dropout rate assumed to be 10%) and the enrollment period was 24 months, with the observation period being 12 months from the date of the enrolling the last patient, it was predicted that about 160 PFS events would be observed and PFS could be estimated with sufficient accuracy that the 95% CI of the median PFS would be within ± 4 months of the actual median PFS. Therefore, the enrollment target for this study was set at 300 patients.

Statistical analyses were performed using SAS software (version 9.4 SAS Institute Inc., Cary, NC, USA).

This study was conducted in compliance with the Declaration of Helsinki and according to the “Japanese ethical guidelines for epidemiological studies”. The study protocol was approved by Institutional Review Boards of all participating medical facilities.

This study was registered with the University Hospital Medical Information Network (UMIN) Clinical Trials Registry in Japan (UMIN000013164).

## Results

From April 2014 to February 2016, 346 Japanese patients with advanced epithelial ovarian cancer/fallopian tube cancer/primary peritoneal cancer were enrolled at 79 member institutions of the Japanese Gynecologic Oncology Group (JGOG). Follow-up was terminated in February 2017 and data were locked in October 2017. Of the 346 patients enrolled, ineligible patients and patients who did not receive bevacizumab were excluded, and the remaining 333 patients were classified as treated patients. They were divided into the primary analysis cohort (*n* = 293) and the exploratory analysis cohort (*n* = 40) (Fig. 1). The reasons for discontinuation of bevacizumab at the time of data cut-off were completion of the planned treatment schedule (34.8%), progression of the primary disease (26.6%), serious adverse events (14.0%), refusal by the patient because of adverse events (5.5%), and refusal by the patient for reasons other than adverse events (3.8%). At data cut-off, bevacizumab was still being administered to 15.0% of the patients. Patient characteristics are listed in Table 1.

**Table 1:** Characteristics of the patients

	Treated patients *n* = 333	Primary analysis cohort *n* = 293	Exploratory analysis cohort *n* = 40
Age			
Median			
(range)	58 (21–83)	58 (27–83)	62 (21–75)
Age ≥ 70			
*n* (%)	51 (15.3)	42 (14.3)	9 (22.5)
ECOG PS^a^			
0			
*n* (%)	274 (82.3)	243 (82.9)	31 (77.5)
1			
*n* (%)	50 (15.0)	44 (15.0)	6 (15.0)
2			
*n* (%)	8 (2.4)	5 (1.7)	3 (7.5)
Unknown			
*n* (%)	1 (0.3)	1 (0.3)	0
Primary site			
Ovary			
*n* (%)	268 (80.5)	234 (79.9)	34 (85.0)
Fallopian tube			
*n* (%)	34 (10.2)	32 (10.9)	2 (5.0)
Peritoneum			
*n* (%)	31 (9.3)	27 (9.2)	4 (10.0)
FIGO stage^b^			
IIIA			
*n* (%)	10 (3.0)	7 (2.4)	3 (7.5)
IIIB			
*n* (%)	36 (10.8)	29 (9.9)	7 (17.5)
IIIC			
*n* (%)	191 (57.4)	168 (57.3)	23 (57.5)
IV			
*n* (%)	96 (28.8)	89 (30.4)	7 (17.5)
Histology^c^			
Serous			
*n* (%)	220 (66.1)	193 (65.9)	27 (67.5)
Clear cell			
*n* (%)	43 (12.9)	36 (12.3)	7 (17.5)
Endometrioid			
*n* (%)	34 (10.2)	31 (10.6)	3 (7.5)
Mucinous			
*n* (%)	9 (2.7)	8 (2.7)	1 (2.5)
Other or not specified			
*n* (%)	27 (8.1)	25 (8.5)	2 (5.0)
Surgery			
PDS			
*n* (%)	224 (67.3)	199 (67.9)	25 (62.5)
NAC → IDS			
*n* (%)	109 (32.7)	94 (32.1)	15 (37.5)
Surgical outcome			
Optimal			
*n* (%)	177 (53.2)	148 (50.5)	29 (72.5)
Suboptimal			
*n* (%)	156 (46.8)	145 (49.5)	11 (27.5)
Bowel resection			
Yes			
*n* (%)	49 (14.7)	37 (12.6)	12 (30.0)
No			
*n* (%)	284 (85.3)	256 (87.4)	28 (70.0)
Platinum-based chemotherapy			
Paclitaxel + carboplatin			
*n* (%)	293 (88.0)	293 (100.0)	0
Docetaxel + carboplatin			
*n* (%)	13 (3.9)	0	13 (32.5)
Dose-dense paclitaxel + carboplatin			
*n* (%)	22 (6.6)	0	22 (55.0)
Other			
*n* (%)	5 (1.5)	0	5 (12.5)
Bevacizumab exposure			
Median no. of cycles			
*n* (range)	17 (1–40)	17 (1–40)	16.5 (1–25)
Median duration			
Month (range)	12.7 (0–29.5)	12.7 (0–29.5)	12.7 (0–20)
Administration delayed by AE			
*n* (%)	185 (55.6)	155 (52.9)	30 (75.0)
Skipped			
*n* (%)	38 (11.4)	35 (11.9)	3 (7.5)
Dose modification			
*n* (%)	3 (0.9)	3 (1.0)	0

*AE* adverse event

^a^Eastern Cooperative Oncology Group (ECOG) performance status (PS)

^b^FIGO staging system 1988

^c^WHO classification 2003

### Safety

**Fig. 2:**
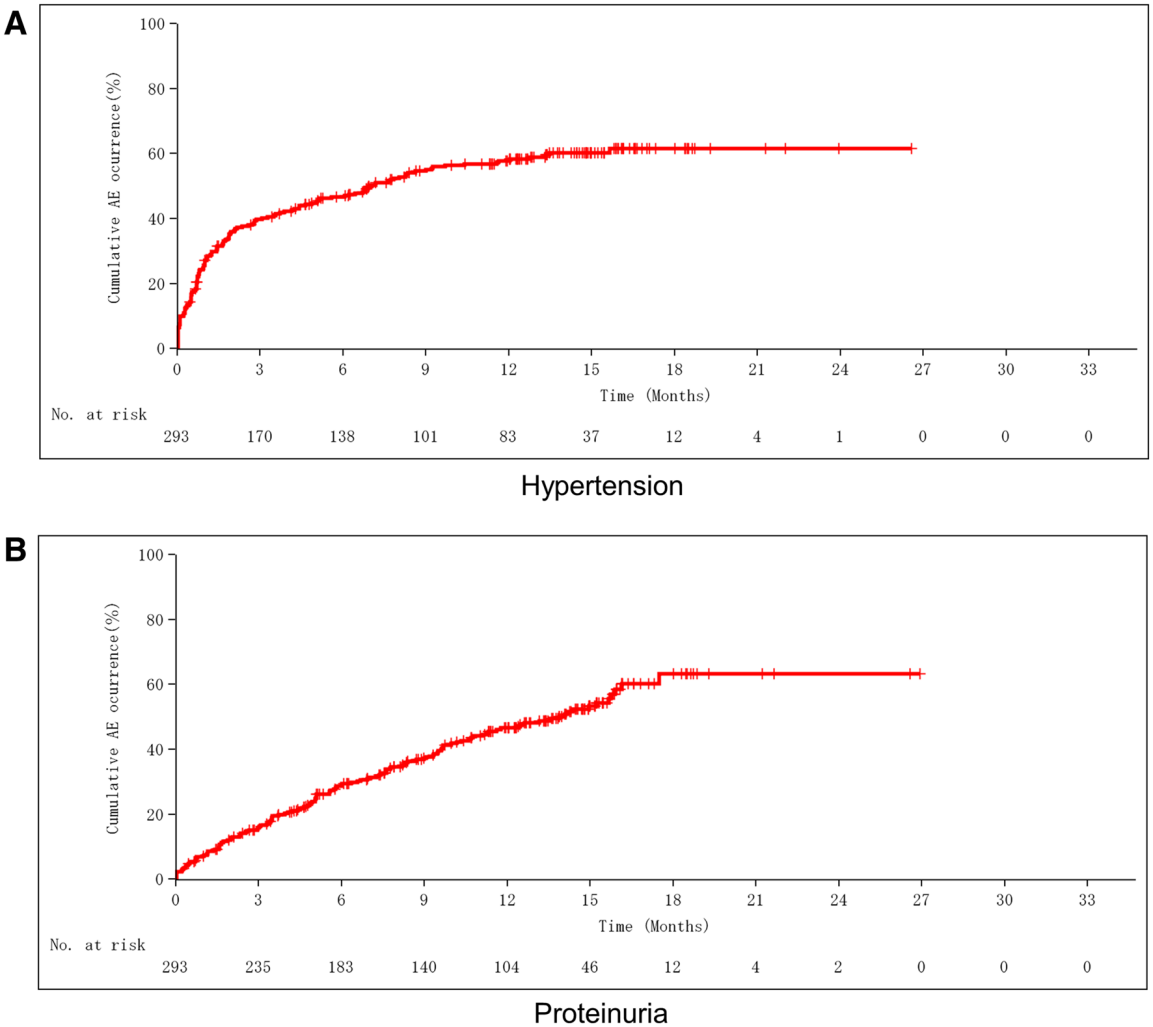
**a** Cumulative number of patients developing hypertension (all grade). **b** Cumulative number of patients developing proteinuria (all grade)

Adverse events in the primary analysis cohort included Grade 2, 3, and 4 hypertension (23.2, 22.9 and 0.3%, respectively), Grade 2 and 3 proteinuria (17.1 and 12.6%), Grade 2 bleeding (2.7%), Grade 2 and 3 thromboembolic events (1.0 and 1.4%), Grade 2 and 3 gastrointestinal perforation (0.3 and 0.3%), Grade 2 wound dehiscence (1.0%), and Grade 3 fistula (0.7%). The incidence rates of bleeding, thromboembolic events, gastrointestinal perforation, wound dehiscence, and fistula were lower in this study than in previous reports, while the incidence rates of hypertension and proteinuria were higher in this study. No patient developed congestive heart failure or posterior reversible encephalopathy syndrome. Grade 3 or more severe neutropenia, febrile neutropenia, anemia, thrombocytopenia, fatigue, malaise, allergic reactions, peripheral neuropathy, and nausea were observed in this study. However, these events and their frequencies were within the known range of adverse events for paclitaxel plus carboplatin (Table 2). None of the 49 patients who received bowel resection developed gastrointestinal perforation or fistula. The incidence rates of bevacizumab-specific adverse events in the primary analysis cohort were compared during combination therapy with bevacizumab and paclitaxel plus carboplatin versus during maintenance bevacizumab monotherapy. This comparison showed that the incidence rate of hypertension was higher during combination therapy and the incidence rate of proteinuria was higher during maintenance therapy, while there was no difference in the incidence rates of gastrointestinal perforation and thromboembolic events between the two treatment periods. Hypertension of any grade occurred in 93 patients (31.7%) before 6 months of bevacizumab treatment and occurred in 73 patients (21.9%) after 6 months of treatment, with the median time to onset being 1.2 months (range 0–16.5 months). Grade 3 or higher hypertension occurred in 41 patients (14.0%) before 6 months of bevacizumab treatment and occurred in 32 patients (9.6%) after 6 months of treatment. In contrast, proteinuria of any grade occurred in 39 patients (13.3%) before 6 months of bevacizumab treatment and occurred in 107 patients (32.1%) after 6 months of treatment, with the median time to onset being 5.0 months (range 0–17.5 months). Grade 3 or higher proteinuria occurred in 8 patients (2.7%) before 6 months of bevacizumab treatment and occurred in 33 patients (9.9%) after 6 months of treatment (Table 3; Fig. 2). Incidence rates of bevacizumab-specific adverse events in the exploratory analysis cohort were similar to those in the primary analysis cohort (Table 4).

**Table 2:** All adverse events in the primary analysis cohort (*n* = 293)

	Grade				
	*n* (%)				
	1	2	3	4	5
Neutropenia	–	–	54 (18.4%)	151 (51.5%)	0
Febrile neutropenia	–	–	12 (4.1%)	2 (0.7%)	0
Anemia	–	–	27 (9.2%)	2 (0.7%)	0
Thrombocytopenia	–	–	30 (10.2%)	5 (1.7%)	0
Fatigue	–	–	6 (2.0%)	0	0
Malaise	–	–	1 (0.3%)	0	0
Allergic reaction	–	–	4 (1.4%)	0	0
Peripheral neuropathy	–	–	10 (3.4%)	0	0
Nausea	–	–	6 (2.0%)	0	0
Hypertension	24 (8.2%)	68 (23.2%)	67 (22.9%)	1 (0.3%)	0
Proteinuria	51 (17.4%)	50 (17.1%)	37 (12.6%)	0	0
Bleeding^a^	3 (1.0%)	8 (2.7%)	0	0	0
Thromboembolic events^b^	2 (0.7%)	3 (1.0%)	4 (1.4%)	0	0
Gastrointestinal perforation	0	1 (0.3%)	1 (0.3%)	0	0
Wound dehiscence	1 (0.3%)	3 (1.0%)	0	0	0
Fistula	0	0	2 (0.7%)	0	0
Congestive heart failure	0	0	0	0	0
Posterior reversible encephalopathy syndrome	0	0	0	0	0

^a^Non-central nervous system (CNS) bleeding

^b^Venous thromboembolism

**Table 3:** Incidence rates of bevacizumab-specific adverse events ≥ Grade 3 in the primary analysis cohort during combined therapy and maintenance therapy

	Paclitaxel and carboplatin + bevacizumab (%)	Bevacizumab maintenance (%)
Hypertension	14.0	9.2
Proteinuria	2.7	9.9
Bleeding^a^	0	0
Thromboembolic events^b^	0.7	0.7
Gastrointestinal perforation	0	0.3
Wound dehiscence	0	0
Fistula	0.3	0.3

^a^Non-central nervous system (CNS) bleeding

^b^Venous thromboembolism

**Table 4:** Incidence rates of adverse events ≥ Grade 3 in the primary analysis cohort and the exploratory analysis cohort

	Adverse events ≥ Grade 3 (%)		
	Treated patients	Primary analysis cohort	Exploratory analysis cohort
Neutropenia	70.0	70.0	70.0
Febrile neutropenia	5.4	4.8	10.0
Anemia	14.7	9.9	50.0
Thrombocytopenia	13.8	11.9	27.5
Fatigue	2.4	2.0	5.0
Malaise	0.3	0.3	0
Allergic reaction	1.2	1.4	0
Peripheral neuropathy	3.9	3.4	7.5
Nausea	1.8	2.0	0
Hypertension	22.2	23.2	15.0
Proteinuria	13.5	12.6	20.0
Bleeding	0	0	0
Thromboembolic events^a^	1.2	1.4	0
Gastrointestinal perforation	0.3	0.3	0
Wound dehiscence	0	0	0
Fistula	0.6	0.7	0
Congestive heart failure	0	0	0
Posterior reversible encephalopathy syndrome	0	0	0

^a^Venous thromboembolism

### Efficacy

**Fig. 3:**
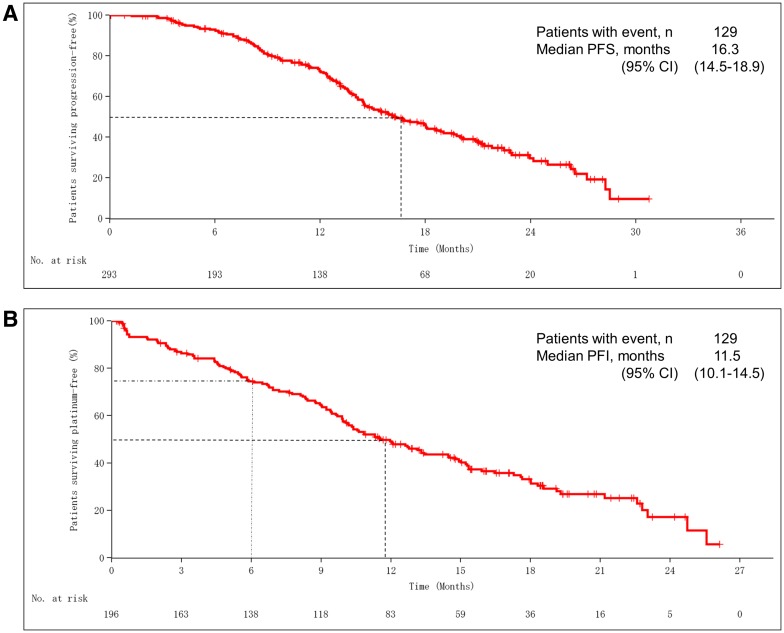
**a** Progression-free survival in the primary analysis cohort. The median progression-free survival (PFS) time was 16.3 months (95% CI 14.5–18.9). **b** Platinum-free interval in the primary analysis cohort. The median platinum-free interval (PFI) was 11.5 months (95% CI 10.1–14.5) and the platinum-resistant recurrence rate (proportion of patients with disease progression after < 6 months) was 24.5%

With regard to efficacy in the primary analysis cohort, median PFS was 16.3 months (95% CI 14.5–18.9) (Fig. 3a). Subgroup analysis of the primary cohort is displayed in Table 5.

**Table 5:** Subgroup analysis of progression-free survival (PFS) in the primary analysis cohort

	PFS					
	*n*	Event	Median (month)	95% CI	1-year PFS rate (%)	95% CI
All patients	293	129	16.3	14.5–18.9	72.5	65.8–78.1
Age						
< 60	157	70	15.5	13.7–21.0	74.4	65.1–81.6
≥ 60	136	59	16.3	14.4–19.0	70.4	60.0–78.5
ECOG PS^a^						
0	243	100	17.5	14.5–20.0	76.3	69.1–82.1
1.2	49	29	14.4	10.9–19.8	56.6	39.7–70.4
FIGO stage^b^						
Stage III	204	81	18.1	15.8–21.0	75.4	67.1–81.8
Stage IV	89	48	14.1	13.0–15.5	67.3	55.0–76.9
Surgery						
PDS	179	65	20.1	15.0–22.8	74.4	65.6–81.3
NAC-IDS	92	51	14.7	13.0–17.9	69.7	57.1–79.3
Surgical outcome						
Optimal	148	50	20.8	16.7–26.5	77.6	67.5–84.9
Suboptimal	145	79	14.1	13.1–15.5	68.7	59.3–76.4
Histology^c^						
Serous	193	78	17.1	14.7–19.8	76.9	68.6–83.2
Endometrioid	31	12	13.1	12.0–NE	64.8	39.7–81.5
Clear cell	36	22	12.3	8.3–15.3	50.5	31.1–67.0

*NE* not estimable

^a^Eastern Cooperative Oncology Group (ECOG) performance status (PS)

^b^FIGO staging system 1988

^c^WHO classification 2003

In 89 patients with measurable lesions, the response rate was 77.5% (95% CI 67.4–85.7). Subgroup analysis stratified by tumor histology showed that the response rate was high in patients with serous and endometrioid carcinoma, but the rate was low in those with clear cell carcinoma and other rare histological types (Table 6).

**Table 6:** Response rate stratified by tumor histology in patients with measurable lesions from the primary analysis cohort

	*n*	Response rate	
		%	95% CI
All cases	89	77.5	67.4–85.7
Serous^a^	60	81.7	69.6–90.5
Endometrioid^a^	10	80	44.4–97.5
Clear cell^a^	11	63.6	30.8–89.1
Others^a^	8	62.5	24.5–91.5

^a^WHO classification 2003

In the primary analysis cohort, the platinum-free interval (PFI) was evaluated for 196 patients who completed platinum-based chemotherapy. It was found that disease progression occurred after < 6 months in 24.5% (resistant), from 6 to < 12 months in 23.5% (partially sensitive), and after ≥ 12 months or not at all in 42.4% (fully sensitive) (Table 7). The median PFI was 11.5 months (95% CI 10.1–14.5) (Fig. 3b).

**Table 7:** Recurrence and platinum sensitivity in the primary analysis cohort (*n* = 196)

	*n*	%
Resistant^a^	48	24.5
Partially sensitive^b^	46	23.5
Fully sensitive^c^	83	42.4
Censored (< 6 months)	10	5.1
Censored (6 to < 12 months)	9	4.6

^a^Progression after < 6 months

^b^Progression from 6 to < 12 months

^c^Progression after ≥ 12 months or no progression

## Discussion

Compared with the incidence rates revealed by previous studies performed in Europe and the United States, including phase 3 clinical studies (GOG-0218 and ICON7) [3, 4] and a large-scale prospective observational study (ROSiA) [11], the incidence rates of gastrointestinal perforation/fistula, thromboembolic events, and bleeding were lower in the present study (JGOG3022), while incidence rates of hypertension and proteinuria were higher. In particular, gastrointestinal perforation and thromboembolic events were markedly less frequent in this study. These results suggest that bevacizumab is relatively safe for Japanese patients with advanced ovarian cancer, if administered according to the inclusion and exclusion criteria used in this study. Additional analysis of data from the GOG-0218 study showed that a history of bowel resection is a risk factor for gastrointestinal perforation [12]. However, 12.6% of patients in the primary analysis cohort and 14.7% of all patients had a history of bowel resection in the present study, and none of them developed gastrointestinal perforation or fistula. Therefore, prior bowel resection does not seem to be a risk factor for gastrointestinal perforation in Japanese patients.

On the other hand, the incidence rate of hypertension was higher in this study than in the GOG-0218 study and was similar to that in the ROSiA study. We found that hypertension occurred earlier after the start of treatment than in the ROSiA study, suggesting that antihypertensive therapy should be started earlier in Japanese patients. Proteinuria may be due to cumulative toxicity and there is no effective treatment, which means it should be managed carefully as a dose-limiting factor. However, hypertension and proteinuria were both tolerable in the present study. Thus, there were differences in the profile and incidence rates of adverse events between Japanese and Western patients that may be due to racial differences, although the reasons are currently unclear. In a previous study of bevacizumab for lung cancer, the incidence rate of hypertension was also higher in Japanese patients than in Western patients [5].

Regarding efficacy, the median PFS was 16.3 months in this study, which was not inferior to either the GOG-0218 study [3] or the GOG-0262 study [13], during which bevacizumab was administered concomitantly with standard treatment using paclitaxel plus carboplatin to similar patient populations as in this study. Since the present study was a single-arm observational study, simple comparison with the results of the phase 3 clinical studies is not appropriate. However, at least we can conclude that concomitant administration of bevacizumab with paclitaxel plus carboplatin is a useful regimen for Japanese patients. The data obtained in this may be considered inadequate because the number of PFS events was lower than expected, but conversely the inhibitory effect on recurrence was greater than expected. In Japan, dose-dense paclitaxel with carboplatin is often selected as first-line therapy for patients with advanced epithelial ovarian cancer. Henceforth, we will be able to choose bevacizumab with paclitaxel plus carboplatin or dose-dense paclitaxel with carboplatin for these patients, by taking into consideration individual background factors and the adverse event profile.

Subgroup analysis showed that PFS was shorter and the response rate was lower in patients with clear cell carcinoma than in patients with serous or endometrioid carcinoma. Many ovarian clear cell carcinomas show intrinsic resistance to platinum-based chemotherapy, which may influence the results of studies [14, 15]. Retrospective studies showed that the response rate of patients with clear cell carcinoma to first-line taxane plus platinum chemotherapy was 20–50% [16], while the only prospective large-scale phase 3 clinical study of first-line chemotherapy for ovarian clear cell carcinoma (JGOG3017/GCIG) revealed that the response rate to paclitaxel and carboplatin was 46.7% in 15 patients with measurable lesions [17]. Although the response rate was low in these studies, we obtained a higher response rate of 63.6% for clear cell carcinoma in the present study. It has already been reported that bevacizumab has potential activity against clear cell carcinoma in vitro [18]. These findings suggest that combining bevacizumab with paclitaxel plus carboplatin increases the anticancer activity against clear cell carcinoma. Among Japanese patients with epithelial ovarian cancer, 23–25% have clear cell carcinoma, which is a significantly larger proportion than among Western patients (5–10%) [12, 17, 19]. This suggests that addition of bevacizumab to paclitaxel plus carboplatin for patients with clear cell carcinoma could be a very important treatment strategy in Japan.

It has been reported that the platinum-resistant recurrence rate is generally 32–40% in patients with advanced epithelial ovarian cancer at their first recurrence [20, 21], while the platinum-resistant recurrence rate was 24.5% in this study. Therefore, it is possible that combining bevacizumab with paclitaxel plus carboplatin could decrease the platinum-resistant recurrence rate. Recurrence eventually affects 70–80% of patients with advanced epithelial ovarian cancer [2, 22], with platinum sensitivity and primary PFI being repowered as prognostic factors for recurrent epithelial ovarian cancer [22, 23]. Furthermore, it is currently reported that the concept of “platinum sensitivity” based on the platinum-free interval may also be applicable to patients who have been previously treated with bevacizumab plus platinum-based chemotherapy [24]. Accordingly, a beneficial decrease of the platinum-resistant recurrence rate and prolongation of PFI could possibly be achieved in clinical practice.

In conclusion, this was the first large-scale prospective study performed in Japan to evaluate the significance of combining bevacizumab with paclitaxel plus carboplatin to treat newly diagnosed advanced epithelial ovarian cancer. The results suggested that bevacizumab is safe and effective in Japanese patients. Combined therapy with bevacizumab could possibly decrease platinum-resistant recurrence and prolong PFI. It may also be more effective for clear cell carcinoma than standard treatment.
